# Efficient cell death mediated by bioengineered killer extracellular vesicles

**DOI:** 10.1038/s41598-023-28306-8

**Published:** 2023-01-19

**Authors:** Julia Dancourt, Ester Piovesana, Gregory Lavieu

**Affiliations:** 1 Université Paris Cité, INSERM U1316, UMR 7057/CNRS, Paris, France; 2 Present Address: Laboratory for Aging Disorders, Laboratories for Translational Research, EOC Bellinzona (Bios+), Bellinzona, Switzerland

**Keywords:** Biotechnology, Cell biology

## Abstract

Extracellular vesicles (EVs) are biological vehicles that are thought to mediate cell–cell communication via the transfer of biomolecules from donor to acceptor cells. Repurposing those natural vesicles into therapeutics delivery vectors is a high priority challenge for translational science. Here we engineer donor cells to produce copious amount of fusogenic EVs loaded with the catalytic domain of the Diphteria Toxin, known to trigger cell death through protein synthesis inhibition. We show that, when incubated with cancer acceptor cells, these Killer EVs block protein synthesis and lead to cell death. This proof of concept establishes the efficacy of Killer EVs in vitro, and suggests that further development may lead to tumor ablation in vivo, expanding the existing cancer therapeutics arsenal.

## Introduction

Extracellular Vesicles, hereafter named EVs, are virtually secreted by all kind of cells/tissues and circulate in bodily fluids^1^. EVs have been associated with a broad spectrum of physiological functions^2^. They contain cargoes such as nucleotides and proteins, which are protected by a lipid bilayer from the extracellular environment, and have been proposed to mediate cargo transfer from acceptor to donor cells. Although the systematic occurrence of EV cargo delivery is still debated, several studies, including ours^3–5^, formally demonstrated that EV cargo release within the cytosol of acceptor cells does occur. Quantification of the process however suggests that EV delivery is a relatively low yield process^4^. Nevertheless, the uptake/delivery capacity of EVs appears to be superior to that of liposomes, commonly used in therapies^6^.

This reinforces the long-standing interest in utilizing and capitalizing on EV properties to develop novel vectors dedicated to therapeutics delivery. This line of research has been extensively investigated, especially towards the delivery of nucleotides^7^. Delivery of protein-based therapeutics through EVs has however been neglected, although several lethal toxins have been the objects of intensive translational research, especially in the context of cell/tissue ablation. This is particularly relevant to the field of cancer, in which those toxins have long been contenders to promote tumor ablation^8,9^.

Diphteria toxin is one of the most potent toxins known and its biology is well characterized^10^. It belongs to the A-B toxin family, for which the B-domain enables cell surface binding, internalization followed by membrane penetration and cytosolic liberation of the catalytic A-domain (DTA), responsible, in the case of DTA, for protein synthesis blockade through inhibition of the elongation factor eIF2. The inhibitory action of DTA on eIF2 occurs through the specific ADP-ribosylation of a diphtamide-modified histidine residue on eIF2^11^. Enzymes DPH1-5 are required for the biosynthesis of diphtamide and depletion of any of these proteins abolishes DTA-sensitivity^12,13^.

Importantly, DTA on its own is not capable of penetrating the cell even at a high concentration in the extracellular media^14^. However, expression of DTA alone in the cytoplasm is sufficient to ultimately kill cells through protein translation blockage. This makes DTA a suitable candidate for EV-mediated delivery.

In the past, liposome-mediated DTA delivery has been tested but led to poor efficiency^15^. More recently, DTA-expressing lentiviruses emanating from DPH-depleted cells have been shown to trigger the death of infected wildtype cells^16^, but lentiviruses come with high safety constraints when considered for therapeutic applications.

Here we tested the capacity of EVs to deliver lethal DTA to acceptor cells, in a virus-free context. First, we engineered DPH2KD donor cells that are DTA-resistant and can thus express detectable amounts of the toxin. Secondly, we added of a palmitoylation motif to DTA, which enabled its reversible membrane anchoring and its efficient loading into EVs, while maintaining its capacity to abolish protein synthesis. Treatment of acceptor cells with DTA-containing EVs show modest but measurable protein synthesis inhibition, that is dose-dependent. When equipped with the VSV-G fusogenic protein, DTA-containing EVs show a five-fold increase in protein synthesis inhibition towards the treated acceptor cells, which led to massive cell death.

This proof of concept demonstrates that Killer EVs are potent in vitro to trigger protein translation inhibition followed by cell death and suggest that the next generation of Killer EVs may trigger specific tumor ablation in vivo.

## Results

### Engineering DTA-containing EVs

**Figure 1:**
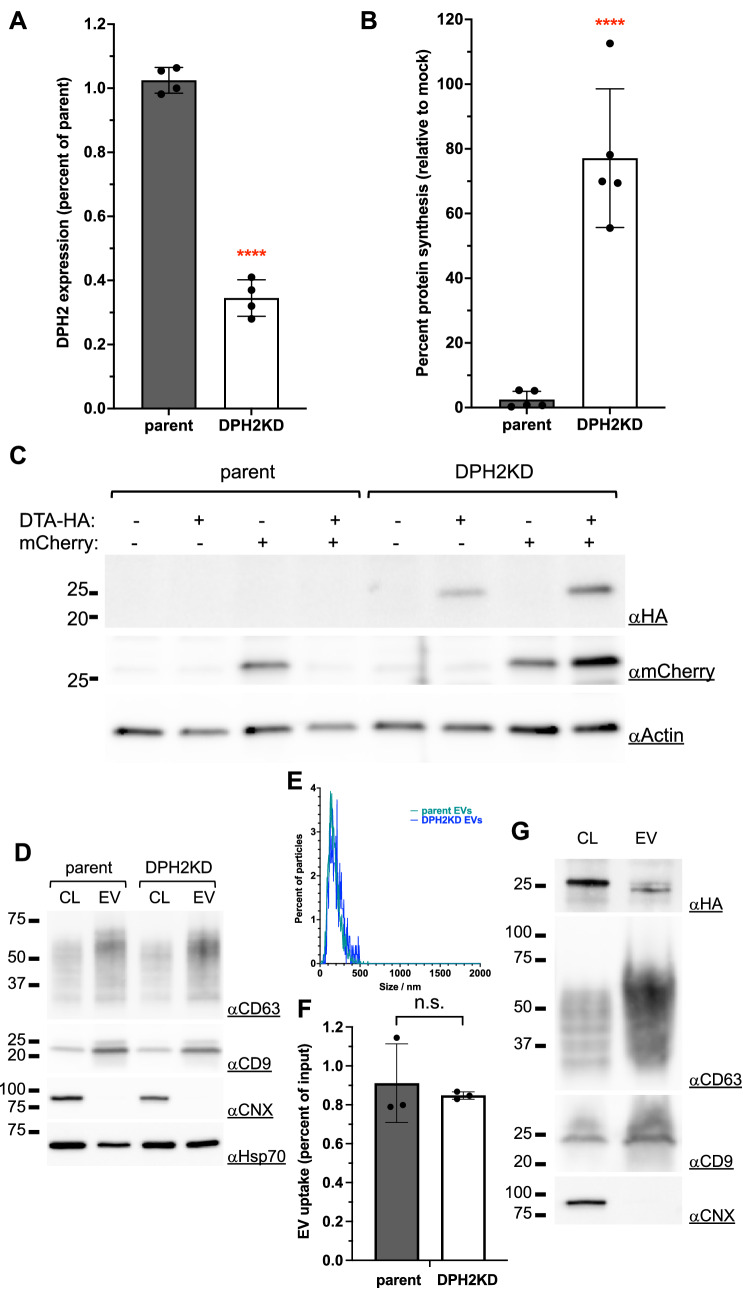
DTA-resistant donor cells. (**A**) Parent Hela cells were infected with a lentivirus encoding a shRNA targeting the DPH2 gene to generate DPH2KD cells. DPH2 knockdown was confirmed by qRT-PCR. ****: *p* < 0.0001. (**B**) Parent or DPH2KD cells were co-transfected with NLuc-Hsp70 along with a mock plasmid or a plasmid encoding DTA-HA. 30 h post-transfection, the percentage of protein synthesis in cells co-transfected with a DTA-HA plasmid was calculated as the percentage of NanoLuciferase activity in these cells relative to mock-transfected cells. ****: *p* < 0.0001. (**C**) A plasmid encoding DTA-HA and/or a plasmid encoding mCherry were transfected into parent or DPH2KD cells. 36 h post-transfection, equal protein amounts of each sample were analyzed by western blot. (**D**) EVs were prepared from parent or DPH2KD cells and analyzed by western blot along with total cell lysates (CL). The same amount of protein was loaded for each sample. (**E**) Particle metrics for EVs in D. (**F**) EVs from NLuc-Hsp70-expressing parent or DPH2KD cells were incubated on acceptor (parent HeLa) cells for 24 h. EV uptake was calculated as percent of input material. (**G**) EVs were obtained from DPH2KD cells transfected with a plasmid encoding DTA-HA. Total cell lysates (CL) and EV proteins were analyzed by western blot. The same amount of protein was loaded for each sample.

Our goal was to generate EV donor cells that would secrete potent DTA-containing EVs. First, in order to express DTA in donor cells, we’d have to render them resistant to the toxin. We thus generated, through RNA interference, stable donor Hela cells knocked down for DPH2, an enzyme responsible for diphtamide synthesis which is absolutely required for DTA sensitivity^12^. DPH2 expression was assessed by qRT-PCR, which showed a 65% (± 5%) knockdown efficiency in the selected DPH2KD cells (Fig. 1A).

To study whether the DPH2KD cells were indeed DTA-resistant, we assessed their protein synthesis activity using a Nanoluciferase (NLuc)-encoding plasmid as a reporter. Parent and DPH2KD cells were co-tranfected with the NLuc-plasmid and with either an empty plasmid (mock) or with a plasmid encoding HA-tagged DTA (DTA-HA). We measured the luminescence activity 30 h post-transfection. Co-transfection with DTA completely abolished NLuc expression within parent cells (less than 3% of control protein synthesis activity), whereas DPHK2KD cells retained more than 75% of protein synthesis activity in the same conditions (Fig. 1B). This demonstrated that DTA-HA expression efficiently blocked protein synthesis in parent cells, and that DPH2KD cells were DTA resistant.

We confirmed these data by co-transfecting parent or DPH2KD cells with plasmids encoding mCherry as a reporter and plasmids expressing either mock or DTA-HA. We assessed protein expression by western blot 36 h post-transfection. The mCherry protein was detected in parent cells when co-transfected with a mock plasmid but was absent from cells co-transfected with DTA-HA (Fig. 1C). Note that DTA-HA is not detectable in those cells either, suggesting that DTA-HA is so efficient at turning off protein synthesis that an undetectable amount of the toxin is enough to inhibit its own expression. Only long-lived proteins such as actin remained detectable in these conditions. On the other end, both mCherry and DTA-HA were detectable in DPH2KD cells when co-expressed (Fig. 1C). This independently confirmed our previous results and demonstrated that DTA-HA could be expressed in detectable amounts in DPKH2KD cells.

Although DPH2 knockdown has been shown to lead to decreased cellular fitness^16^, we showed here that these cells are able to produce natural EVs that are similar to EVs produced from parent cells, both in terms of composition and size (Fig. 1D, E). Moreover, the EVs produced by DPH2KD cells are as uptake-competent as wildtype EVs (Fig. 1F).

Since DTA-HA could be expressed in DPH2KD cells (Fig. 1C), we hypothesized that it could be passively loaded into EVs. Indeed, it has been shown that abundant endogenous cytosolic protein such as actin or chaperones (such as Hsp70, Fig. 1D), as well as overexpressed cytosolic proteins can be found in EVs^17^. We therefore tested the presence of DTA-HA in EVs emanating from DTA-HA-transfected DPH2KD cells. 36 h post transfection, we isolated EVs through sequential centrifugation, and tested their contents by western blot. Although the EV fraction was enriched in classical EV markers (Hsp70, high molecular weight CD63 and CD9) and depleted of an endoplasmic reticulum marker (Calnexin), suggesting efficient and specific EV isolation, DTA-HA could not or only barely be detected (Fig. 1G). We concluded that DTA-HA was not passively loaded into EVs (even depleted when compared to total cell lysate), and that we needed to increase its loading efficiency.

**Figure 2:**
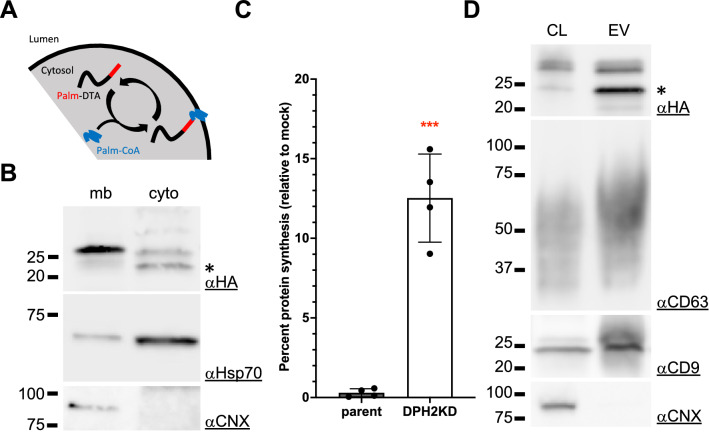
Palm-DTA loading into EVs. (**A**) Scheme of Palm-DTA association with membranes. (**B**) DPH2KD cells were transfected with a plasmid encoding Palm-DTA-HA and subjected to cytosol / membrane fractionation 36 h after transfection. *: de-palmitoylated form of the protein. (**C**) Parent or DPH2KD cells were co-transfected with NanoLuc-Hsp70 along with a mock plasmid or a plasmid encoding Palm-DTA-HA. 30 h post-transfection, the percentage of protein synthesis in cells co-transfected with a Palm-DTA-HA plasmid was calculated as the percentage of NanoLuciferase activity in these cells relative to mock-transfected cells. ***: *p* < 0.001. (**D**) EVs were prepared from DPH2KD cells transfected with a plasmid encoding Palm-DTA-HA. Total cell lysates (CL) and EV proteins were analyzed by western blot. The same amount of protein was loaded for each sample.

Myristoylation and palmitoylation consensus signals are often used to force cargo loading into EVs through membrane association^18,19^. While palmitoylation is a reversible process (Fig. 2A), myristoylation is not and seems incompatible with putative downstream EV-mediated delivery of such a modified cargo. We therefore reasoned that fusing a well-characterized palmitoylation motif^20^ to DTA-HA might better serve our purposes. We therefore engineered a Palm-DTA-HA encoding plasmid and first tested if this protein was prominently associated with membranes. DPH2KD cells expressing Palm-DTA-HA were first submitted to mechanical disruption, and cytosolic and membrane fractions were separated by ultracentrifugation. Palm-DTA-HA was enriched in the membrane fraction (Fig. 2B), which also contained the endoplasmic reticulum marker calnexin, but was strongly depleted of HSP70, a cytosolic protein. On the contrary, the cytosolic fraction, highly enriched in HSP70, but depleted of calnexin, only contained a very low amount of Palm-DTA-HA, as well as a smaller species, probably representing de-palmitoylated Palm-DTA-HA (marked with an asterisk in Fig. 2B). Such a palmitoylation-induced size shift have been shown for other proteins^20,21^. In the absence of a palmitoylation signal, DTA-HA is exclusively found in the cytosolic fraction (Fig. S1A). This validated the membrane association of Palm-DTA-HA.

We then tested the efficacy of Palm-DTA-HA towards protein synthesis inhibition to ensure that the motif did not alter the toxin activity. Using the aforementioned luminescent assay, we showed that Palm-DTA-HA completely abolished protein expression within parent cells (less than 0,3% of control activity), whereas DPH2KD cells showed significant resistance as they retained more than 12% of control activity (Fig. 2C). Note that Palm-DTA-HA resistance of DPH2KD cells was lower than the one for soluble DTA-HA. This suggests that Palm-DTA-HA had a higher toxin potency in our system, probably due to higher expression levels in cells (DTA-HA and Palm-DTA-HA are expressed from different plasmid backbones). This could also be due to membrane anchoring that may stabilize the protein. Moreover, this difference in toxin potency between DTA-HA and Palm-DTA-HA has little impact on donor cell viability at the time of conditioned medium harvest for EV preparation (Fig. S1B). EV preparation was indeed performed from cells that are > 80% viable. This observation is obviously not a handicap, is fully compatible with our goals, and might even serve our interests by enhancing the impact on acceptor cells. It however means that, in its current form, this system requires continuous transfection of new cells for EV production.

Next, we tested if Palm-DTA-HA was efficiently loaded into EVs. DPH2KD cells were transfected with Palm-DTA-HA and conditioned medium was harvested 36 h post-transfection. EVs were, once again, isolated through sequential ultracentrifugation. The EV fraction was positive for HSP70, high molecular weight CD63 and CD9 and negative for Calnexin, as expected. Satisfyingly, we found that Palm-DTA-HA was efficiently loaded into EVs (Fig. 2D). Interestingly, we note that the de-palmitoylated form of Palm-DTA-HA is consistently more prominent in EVs than in the cell lysate (although to varying degrees, Figs. 2D and S1C), suggesting that net de-palmitoylation occurs after EV loading.

### *Testing the potency of DTA-containing EVs *in vitro

**Figure 3:**
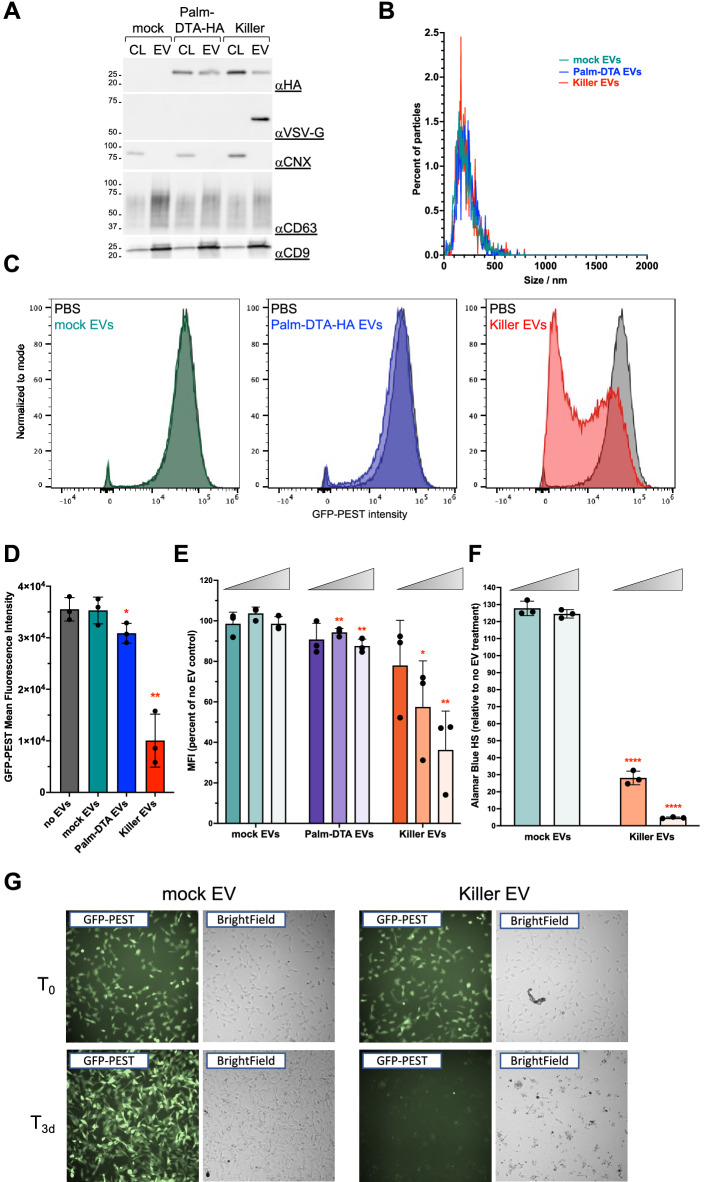
Killer EVs are potent in vitro. (**A**) Western blot characterization of EVs generated from DPH2KD cells transfected with plasmids encoding either Palm-DTA-HA, Palm-DTA-HA + VSV-G (Killer EVs), or a mock plasmid. The same amount of protein was loaded for each sample. (**B**) Particle metrics obtained for the EVs in A. (**C**) 100 µg/mL of the indicated EVs (or DPBS) were incubated for 24 h on GFP-PEST-expressing HT1080 cells. After incubation, green fluorescence was analyzed by FACS and data for each EV-treated sample was plotted against DPBS-treated cells. (**D**) Quantification of data obtained as in panel C. Plotted are the geometric mean of green fluorescence intensity (MFI) for each sample, each data point represents an independent experiment (including independent EV preparation). *: *p* < 0.1; **: *p* < 0.01. (**E**) 10, 20, or 50 µg/mL of the indicated EVs (or DPBS) were incubated for 24 h on GFP-PEST-expressing HT1080 cells. After incubation, cells were analyzed by FACS and the geometric mean of green fluorescence intensity for each sample was obtained and plotted as the percentage of the one obtained for DPBS-treated cells (set at 100%). Each data point represents an independent experiment (including independent EV preparation). *: *p* < 0.1; **: *p* < 0.01. (**F**) A cell viability assay was performed on GFP-PEST-expressing HT1080 cells incubated with the indicated EVs (20 or 50 µg/mL) for 3 days. ****: *p* < 0.0001. (**G**) Microscopic observation of GFP-PEST-expressing HT1080 acceptor incubated with 100 µg/mL Killer EVs indicates a total loss of cells after 3 days.

With our new tools in hand, we decided to test the potency of Palm-DTA-HA-containing EVs on acceptor HT1080 cells, a well-established experimental model that is genetically less aberrant than HeLa cells and highly compatible with future hypothetical genetic screening. To assess the protein synthesis activity in these acceptor cells, we stably equipped them with GFP-PEST^22^, a short half-life version of the Green Fluorescent Protein that gives adequate sensitivity and time resolution and enables single-cell studies by flow cytometry. We previously demonstrated that EV content delivery is a low yield process^4^ and anticipated that natural Palm-DTA-HA-containing EVs might be too limited to dramatically impact protein synthesis in acceptor cells. We therefore decided to also generate, in parallel, Palm-DTA-HA-loaded EVs decorated with VSV-G, a viral fusogen, known to dramatically increase EV content delivery through fusion with the endosomal membranes^23^. Although there is a semantic debate whether VSV-G-bearing EVs should still be called EVs (they have been called Virus-Like Particles, gesicles, gectosomes in the literature), we chose to keep this denomination below as they were found to be indistinguishable by size or marker content from regular EVs (Fig. 3A,B).

We isolated EVs from mock-transfected DPH2KD cells, or DPH2KD cells expressing Palm-DTA-HA with or without VSV-G. EVs were isolated again through sequential ultracentrifugation and characterized by western blot (Fig. 3A) and nanoparticle tracking (Fig. 3B). All EVs showed similar profiles in terms of marker protein content and size distribution. Note that VSV-G is so enriched in EVs that it is barely detectable in total cell lysates.

We then showed that our GFP-PEST-expressing HT1080 clonal cell line would allow us to monitor a putative effect of Palm-DTA-HA EVs on protein synthesis. We showed that, in these cells, green fluorescence has a half-life of about 4,5 h (compared to more than 24 h for wildtype GFP^24^ (Fig. S1C,D), and that these cells are sensitive to DTA activity. Indeed, 24 h of transfection of DTA-HA drastically reduces green fluorescence intensity, as measured by FACS (Fig. S1E,F).

Next, we investigated the effect of EV treatment on these cells. GFP-PEST expression level of untreated acceptor cells was assessed by FACS and compared with signals from cells treated for 24 h with a single dose of either mock EVs, Palm-DTA-HA-containing EVs, or Palm-DTA-HA-VSVG^+^ EVs (hereafter named Killer EVs). Palm-DTA-HA EVs triggered a modest but detectable decrease of GFP-PEST signal (about 15% when considering the mean fluorescence intensity, Fig. 3C,D). Killer EVs, on the other hand, triggered a massive decrease of GFP-PEST signal within acceptor cells (more than 75% when considering the mean fluorescence intensity, Fig. 3C,D). Importantly, we established that Palm-DTA-HA-mediated protein synthesis inhibition is dose dependent for both types of EVs, with again a superior effect of Killer EVs (Fig. 3E). To evaluate cell death induced by Killer EVs over time, we performed a quantitative viability assay on acceptor cells and observed that, after 3 days of incubation with a single dose of EVs, Killer EVs led to important (> 90%), dose-dependent, cell death (Fig. 3F). Moreover, we monitored acceptor cells by fluorescence microscopy in the same conditions and observed that mock EVs-treated cells expanded and maintained their fluorescent signal, while viable acceptor green cells were virtually absent in samples treated with Killer EVs (Fig. 3G). This establishes the lethal potency of Killer EVs on our GFP-PEST-expressing HT1080 clonal cell line. To test whether this effect was transposable to other acceptor cell lines, we performed a cell viability assay on HeLa cells treated with either mock EVs, or Killer EVs for 3 days. Results showed that Killer EVs also had a dose-dependent killing activity on HeLa acceptors (Fig. S1H). This suggests that Killer EVs could be potent on multiple types of acceptor cells.

## Discussion

The aim of this study was to engineer toxin-loaded EVs that would efficiently kill target cells, we named them Killer EVs. We succeeded by (a) engineering EV donor cells that are toxin-resistant and thus can express high amounts of it; (b) engineering an active toxin that is efficiently loaded into EVs; (c) engineering fusogenic EVs to increase their content delivery into acceptor cells.

We chose DTA as a toxin candidate because of its known exceptional potency^14^ and its well-characterized mode of action^10^. Although in vitro DTA-resistance of DPH2KD cells has been characterized before; we further investigated these cells here and observed that they were able to produce EVs that showed most hallmarks of the natural EVs produced in parent cells, including similar uptake efficiency (Fig. 1).

In order to efficiently load DTA into EVs, we appended a palmitoylation signal to its N-terminus (Fig. 2). Although we cannot say whether it’s the palmitoylated, membrane-bound, form of the protein that is active or the reversibly-formed de-palmitoylated, cytosolic form, we showed that Palm-DTA-HA retains DTA’s ability to inhibit protein translation.

The fact that Palm-DTA-HA EVs were able to have an effect, although modest, on acceptor cells suggest that natural EVs deliver their content into the cytoplasm of acceptor cells to a detectable level, which has been debated over recent years. Nevertheless, expressing the VSV-G fusogen on Palm-DTA-HA EVs dramatically increase (fivefold increase) their content delivery into acceptor cells, leading to protein synthesis inhibition and cell death (Fig. 3).

All the EVs produced in this study are expected to be an heterogenous mix including exosomes, ectosomes and exomeres. Although careful characterization of Killer EVs will be needed in the future to better their development, the scope of this proof-of-concept study remains to show their efficiency.

For this study, we chose to work with a heterologous system where the EV donor cells (derived from HeLa) and the EV acceptor cells (derived from HT1080) are of different origin. This was motivated by the will to show the flexibility of the system, but in the future, one can envision using a homologous system, which may or may not be more efficient, but which may be better tolerated in the context of autologous EV therapy for instance. With this in mind, we validated that at least the HeLa donor / HeLa acceptor homologous system is efficient (Fig. S1H).

Our Killer EVs are not artificially targeted, which again has the advantage of flexibility, but also the disadvantage of possible high toxicity as they potentially target all cells indiscriminately. If our Killer EVs were to be used for tumor ablation, one could envision intra-tumoral injection. EVs have indeed been shown to passively target tumor cells through the Enhanced Permeability and Retention (EPR) effect^25^. In the near future, we however plan to equip a new version of Killer EVs with targeting signals and/or other fusogens to increase specificity and tolerability of these new potential therapeutics. VSV-G has indeed been shown to trigger rejection response to lentiviral therapeutics^26^. Humanized fusogens could be envisioned instead.

Finally, EVs seem like an ideal candidate for DTA delivery. DT has indeed long been considered for tumor or tissue ablation^27^, in the form of immunotoxins or through other vehicles (liposomes, viruses), but a few hurdles impeded the success of these endeavors (production, purification, formulation, administration route, immunogenicity). One of the issues related to immunotoxins is the preexisting DT immunity due to existing immunization schemes (> 80% babies in the world have been vaccinated in 2020). EV-mediated delivery of DTA would avoid this issue. Moreover, EV-encapsulated DTA would also be protected from circulating decoy exosomes^28^. EVs also represent a more natural toxin delivery system^29^ than liposomes or viruses, thus less prone to rejection.

The results presented here demonstrate the potency of killer EVs and suggest that further development involving specific targeting and humanized fusogens may lead to novel treatments towards tumor or specific cell/tissue ablation.

## Material and methods

### Cell culture

HeLa and HT1080 cells (ATCC, Virginia, U.S.A.) and their transgenic derivatives were grown in DMEM medium (Gibco, Illinois, U.S.A.) complemented with 10% heat-inactivated Fetal Bovine Serum (Biowest, France) at 37 °C under 5% CO2 and high humidity. HT1080 cells medium was further complemented with MEM NEAA (Gibco, Illinois, U.S.A.).

Stable DPH2KD HeLa cells were obtained by lentiviral transduction of a shRNA targeting DPH2 (Horizon Discovery, Cat # VGH5518-200,302,258, U.K.) and selected with 4 µg/mL puromycin (Gibco, Illinois, U.S.A.). A stable GFP-PEST HT1080 clone was obtained by selecting cells with 0,5 mg/mL geneticin (Gibco, Illinois, U.S.A.) after transfection with a GFP-PEST encoding plasmid (Addgene, Cat # 26,821, Massachusetts, U.S.A.).

Transient transfections were performed using Lipofectamine 2000 (Invitrogen, Massachusetts, U.S.A.) according to the manufacturer’s instructions.

### Plasmid constructs

Plasmids used in this study are summarized in Table S1.

To construct the plasmid encoding DTA-HA, the sequence for DTA (obtained from Addgene, Cat # 42,521, Massachusetts, U.S.A.) was fused to the HA-tag sequence using the Infusion cloning strategy (Takara Bio Europe, France) with XbaI/SpeI cloning sites into a pC4Rhe backbone (ARIAD Pharmaceuticals, Massachusetts, U.S.A.). The DTA-HA construct was then subcloned into a pCDNA3.1 backbone (Invitrogen, Massachusetts, U.S.A.) using NheI/BamHI cloning sites.

To construct the plasmid encoding Palm-DTA-HA, the SNAP25 palmitoylation sequence^20^ was inserted at the N-terminus of DTA-HA using Infusion cloning (Takara Bio Europe, France).

### Cell viability assay

Cells were incubated for 2 h in Alamar Blue HS (Invitrogen, Massachusetts, U.S.A.) and the fluorescence signal was measured according to the manufacturer’s instructions using the iD3 SpectraMax microplate reader (Molecular Devices, California, USA). For cell growth curves, cells were incubated with Alamar Blue HS at the indicated timepoints.

### qRT-PCR

Total RNA was extracted from cells using the Nucleospin RNA kit (Macherey Nagel, France) according to the manufacturer’s instructions. Equal amounts of total RNA were reverse transcribed using the iScript cDNA synthesis kit and subjected to qPCR using the iTaq SYBR green kit (Bio-Rad, France), all following the manufacturer’s instructions. The following primers were used: *PGK*: Forward 5’-AGCTGCTGGGTCTGTCATCCT-3’, Reverse 5’-TGGCTCGGCTTTAACCTTGT-3’; *DPH2*: Forward 5’-CGTGCTTCGTCAACGTTCTG-3’, Reverse 5’-TGGGTTCTGGGCCTCAAA-3’. qPCR was performed in a CFX96 system (Bio-Rad, France) at 95 °C for 10 min, followed by 40 cycles at 95 °C for 15 s, 60 °C for 30 s, and 72 °C for 30 s. DPH2 gene expression was normalized to the PGK housekeeping gene according to the 2-ΔΔCt formula.

### Protein synthesis assay

Parental or DPH2KD HeLa cells were seeded in 24 well plates before being co-transfected with plasmids encoding NLuc-Hsp70 and plasmids encoding either mock, or DTA-HA, or Palm-DTA-HA. 6 h after transfection, cells were detached and split in triplicate wells of a 96 well plate. 24 h later, cells were washed with DPBS and NanoLuc activity was measured in each well using the Nano-Glo Live Cell Assay System (Promega, Wisconsin, USA) following the manufacturer’s instructions using the iD3 SpectraMax microplate reader (Molecular Devices, California, USA). The percentage of protein synthesis was calculated relative to the mock-transfected cells (mock set at 100%) for each cell type tested.

### EV preparation

EV donor cells were transfected with the indicated plasmids for 16 h before being incubated in serum-free DMEM for 20 h. Conditioned medium was harvested and submitted to a 2000 × g centrifugation for 20 min at 4 °C to remove cell debris, and then to a 100,000 × g ultracentrifugation for 1 h 30 at 4 °C (45Ti rotor and Optima XE-90 ultracentrifuge, Beckman Coulter, California, USA) to pellet EVs. The EV pellet was washed with DPBS and centrifuged 1 h 30 at 100,000 × g 4 °C (MLA 50 rotor and Optima MAX-XP ultracentrifuge, Beckman Coulter, California, USA). The washed pellet was resuspended in DPBS and EVs were either stored at -20 °C (if destined to western blot or particle metrics analysis) or immediately applied on acceptor cells.

### EV uptake assay

EVs were obtained from parent or DPH2KD HeLa cells transfected with a plasmid encoding NLuc-Hsp70. Acceptor parent HeLa cells were seeded in 96 well plates the day before EV incubation. The same protein amount of EVs for each condition is incubated with acceptor cells for 24 h. After incubation, acceptor cells are washed twice in DPBS and NanoLuc activity was measured in each well using the Nano-Glo Live Cell Assay System (Promega, Wisconsin, USA) following the manufacturer’s instructions using the iD3 SpectraMax microplate reader (Molecular Devices, California, USA). EV uptake is shown as percent of input material. Statistical significance is obtained through an unpaired t-test.

### Western blot

Cells to be analyzed were scraped on ice in DPBS and pelleted at 1000 × g for 5 min at 4 °C. Cell pellets were resuspended in PBX lysis buffer (DPBS, Triton-X-100 1%, EDTA-free protease/phosphatase inhibitor cocktail (Roche, Switzerland)) and incubated on ice for 10 min with intermittent vortexing. Samples were then submitted to a 15,000 × g centrifugation for 10 min at 4 °C to pellet nuclei and unbroken cells. Supernatants (cell lysates, CL) were collected. Protein concentration of cell lysate and EVs were obtained using the Micro BCA Protein Assay kit (Thermo Scientific, Illinois, USA). Samples were mixed with Laemmli buffer (Bio-Rad, France) containing 10% β-mercaptoethanol, except for CD63, and CD9 detection (no β-mercaptoethanol) and loaded on 4–15% polyacrylamide gels (Bio-Rad, France). After electrophoresis, proteins were transferred on PVDF membranes using the Trans-Blot Turbo system (Bio-Rad, France). Membranes were incubated with DPBS containing 0.05% Tween20 and 5% non-fat milk (blocking buffer) for 30 min at room temperature, then with a 1/1000 dilution of primary antibody (α-Actin (Cat # MAB1501, Millipore, Germany), α-Calnexin (Cat # ab133615, Abcam, U.K.), α-CD63 (Cat # 556,019, BD Bioscience, New Jersey, U.S.A.), α-CD9 (Cat # cbl162, Millipore, Germany), α-Hsp70 (Cat # ADI-SPA-810-D, Enzo LifeScience, New York, U.S.A.), α-HA (Cat # 3724, Cell Signaling, Massachusetts, U.S.A.), α-mCherry (Cat # 5993, BioVision, California, U.S.A.)) in blocking buffer overnight at 4 °C. Membranes were then washed and finally incubated with a 1/5000 dilution of HRP-coupled secondary antibody (α-mouse or α-rabbit, Cat # 115-035-003, Jackson ImmunoResearch, U.K.) in DPBS containing 0.05% Tween20 for 1 h at room temperature. The HRP signal on membranes was developed using the Clarity Western ECL substrate (Bio-Rad, France) and imaged using the ImageQuant LAS 4000 (GE Healthcare Life Sciences, France).

### Cytosol/membrane fractionation

Cells to be analyzed were scraped on ice in DPBS and pelleted at 1000 × g for 5 min at 4 °C. Cell pellets were resuspended in 5 volumes of a hypotonic lysis buffer (10 mM Tris–HCl pH 8, 0,5 mM MgCl_2_ and EDTA-free protease/phosphatase inhibitor cocktail (Roche, Switzerland)) and incubated on ice for 10 min before being homogenized with 10 up-and-down passages through a 26 g needle. Tonicity was restored by the addition of 0,25 volume of the hypotonic buffer containing 0.6 M NaCl. Nuclei and unbroken cells were pelleted at 500 × g for 5 min at 4 °C. EDTA was added to the supernatant to a final concentration of 0.05 M before subjecting the samples to ultracentrifugation at 100,000 × g for 30 min at 4 °C (MLA 80 rotor and Optima MAX-XP ultracentrifuge, Beckman Coulter, California, USA). The resulting supernatant constituted the cytosolic fraction. The pellet was resuspended in PBX and centrifuged at 10,000 × g for 15 min at 4 °C to pellet insoluble material. The supernatant constituted the membrane fraction.

### Particle metrics

Nanoparticle Tracking Analysis was performed using the ZetaView® QUATT (Particle Metrix, Meerbusch, Germany) and its corresponding software (ZetaView 8.02.28). For the size measurements, the 448 nm laser in scatter mode was used. The instrument settings were 25 °C, sensitivity of 80, shutter of 100 at a frame rate of 30 frames per second. 1 mL of sample, diluted in DPBS, was loaded into the cell, and the instrument measured each sample at 11 different positions throughout the cell (1 cycle per position). Image evaluation was done on particles with a minimum brightness of 30, a minimum area of 10 and a maximum area of 1000. Tracelength was set at 15. After automated analysis of all positions and removal of any outlier positions, the size distribution of the particles was obtained.

### FACS analysis

After treatments, cells were detached from cell culture plates with 0,05% Trypsin–EDTA and washed once in DPBS through centrifugation at 1000 × g for 5 min at 4 °C. Cells were finally resuspended in DPBS and kept on ice (less than one hour) until analyzed on an Attune NxT flow cytometer (Thermo Scientific, Illinois, USA). Each sample was incubated with 10 µg/mL DAPI (Merck Millipore, Massachusetts, U.S.A.) right before analysis. The gating strategy is depicted in Fig. S1C. Data was analyzed using the FlowJo software (BD Bioscience, New Jersey, U.S.A.).

### Microscopy

Live cells were imaged with a CellInsight—CX7-LZR (ThermoFisher, Illinois, USA), with a 20X objective. Image analysis was performed using the ImageJ software (NIH, Maryland, U.S.A.).

### Statistical analysis

Unpaired, two-tailed t-test were performed using the Prism software (GraphPad, San Diego, California U.S.A.) on biological replicates. *: *p* < 0.1; **: *p* < 0.01; ***: *p* < 0.001; ****: *p* < 0.0001; n.s.: non statistically significant.

## Supplementary Information

Supplementary Information 1.

Supplementary Information 2.

Supplementary Information 3.

Supplementary Information 4.

## Data Availability

The authors declare that the data supporting the findings of this study are available within the Supplementary information files. Further requests should be addressed to the corresponding author.
