# Supplementary material for: Efficient cell death mediated by bioengineered killer extracellular vesicles
Source: Sci Rep. 2023 Jan 19;13:1086. doi: 10.1038/s41598-023-28306-8 (PMC9852484; doi:10.1038/s41598-023-28306-8)

Figure 1C

Same blot was sequentially incubated with 3 different antibodies, in the indicated order. Raw brightfield (with colored protein molecular weight marker), chemiluminescence images as well as overlays are shown. The red box indicates the part of the blot included in the main figure.

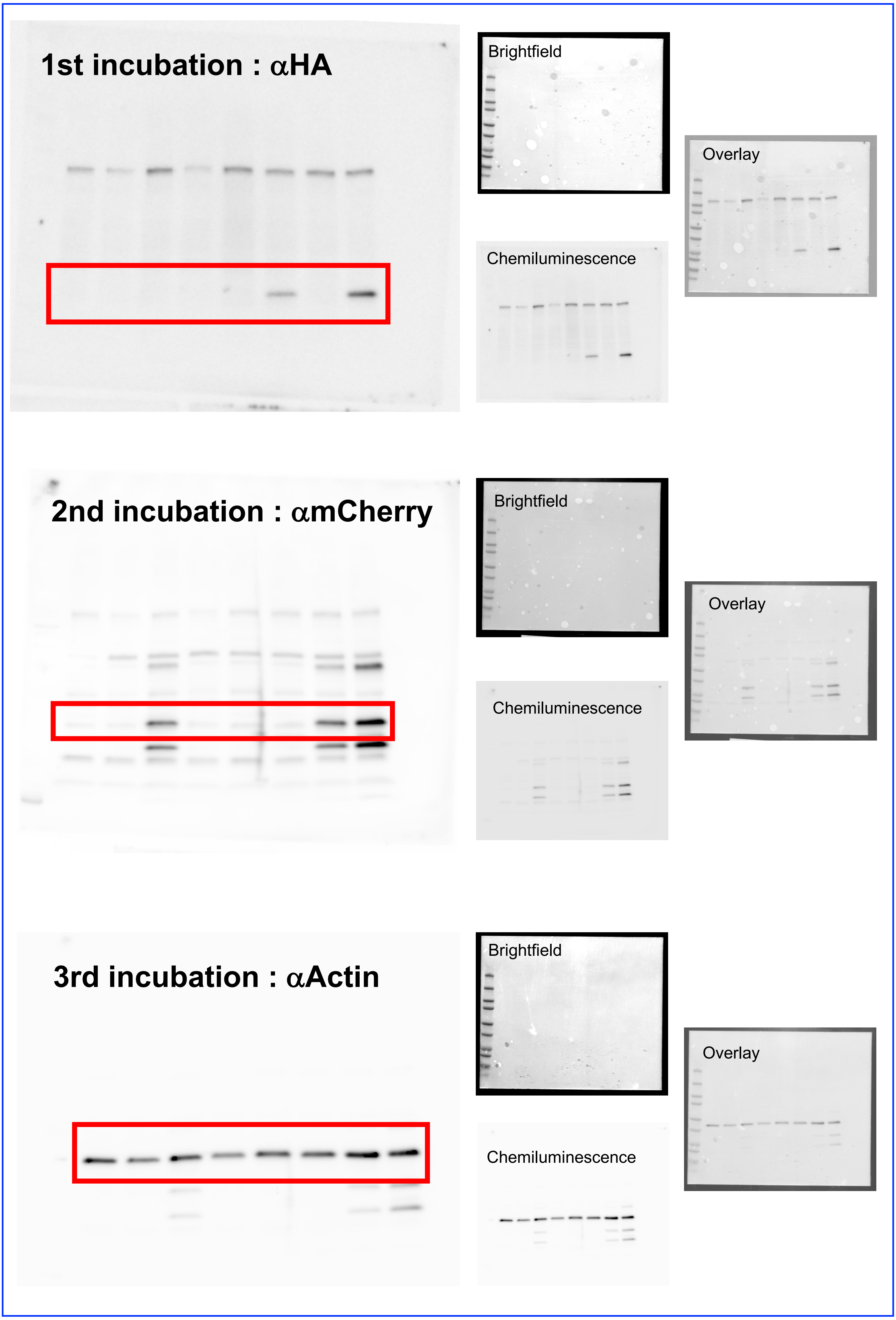

Figure 1D

2 blots were sequentially incubated with 2 different antibodies, in the indicated order. Raw brightfield (with colored protein molecular weight marker), chemiluminescence images as well as overlays are shown. The red box indicates the part of the blot included in the main figure.

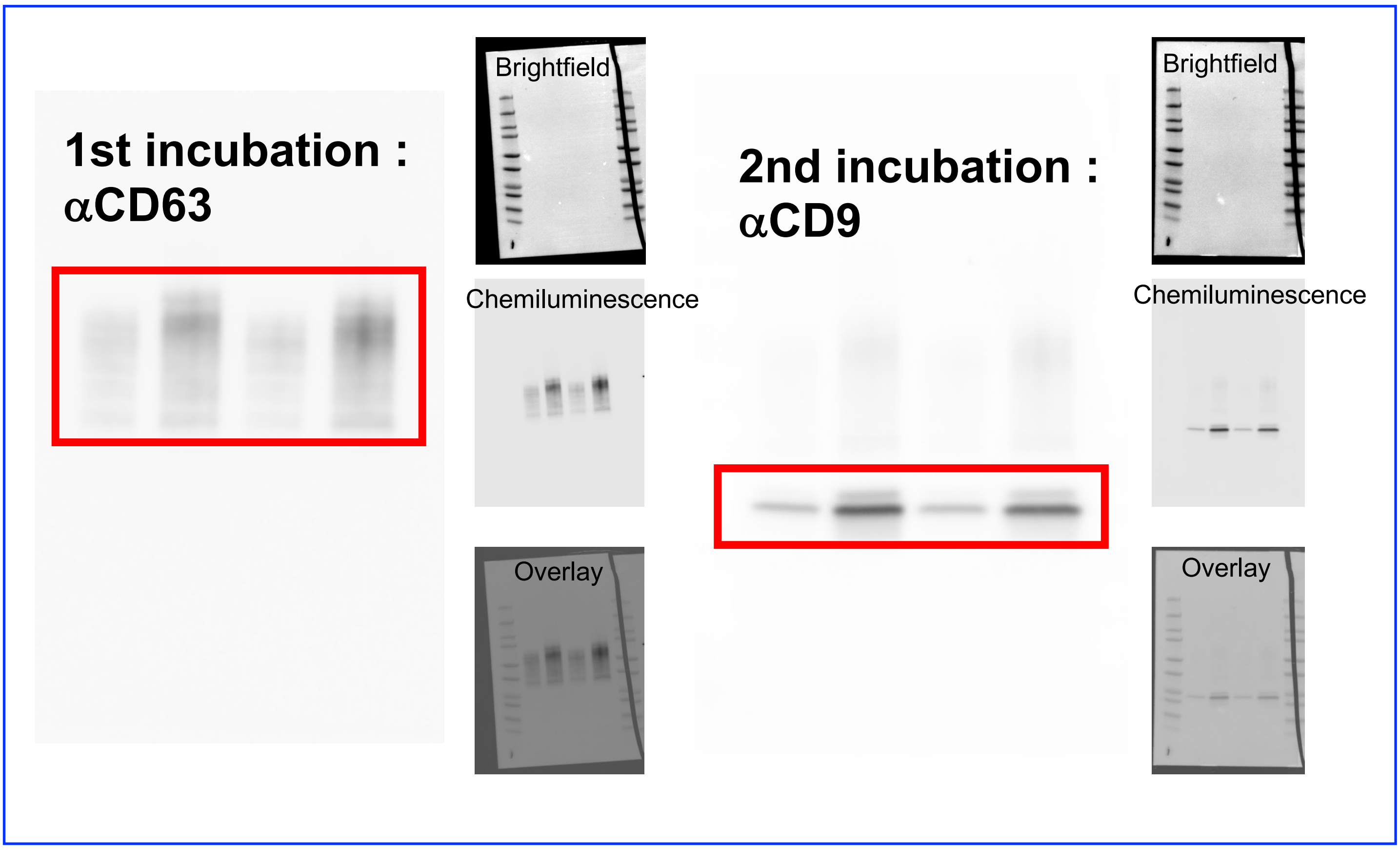

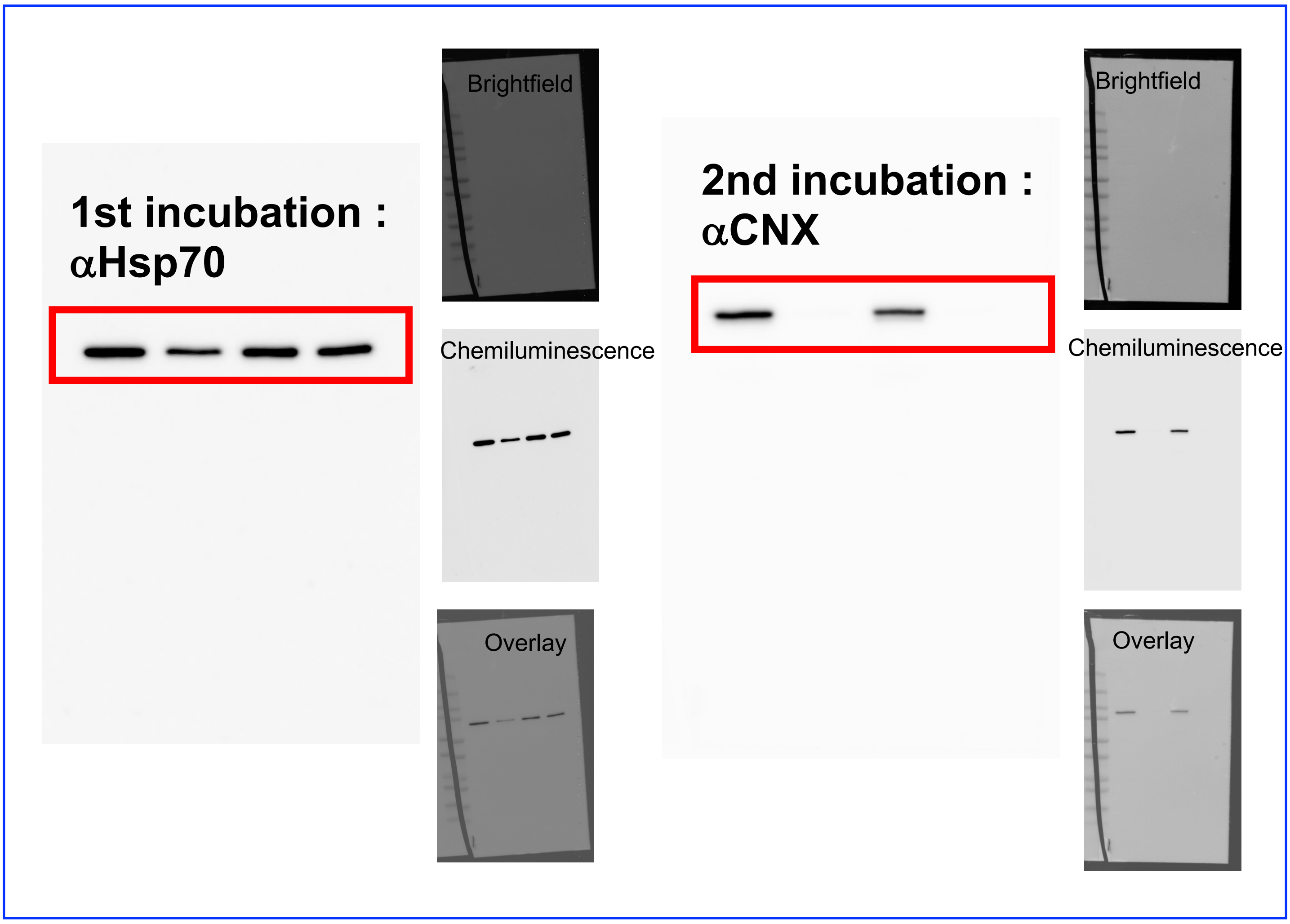

Figure 1G

3 blots were sequentially incubated with one or 2 different antibodies, in the indicated order. Raw brightfield (with colored protein molecular weight marker), chemiluminescence images as well as overlays are shown. The red box indicates the part of the blot included in the main figure.

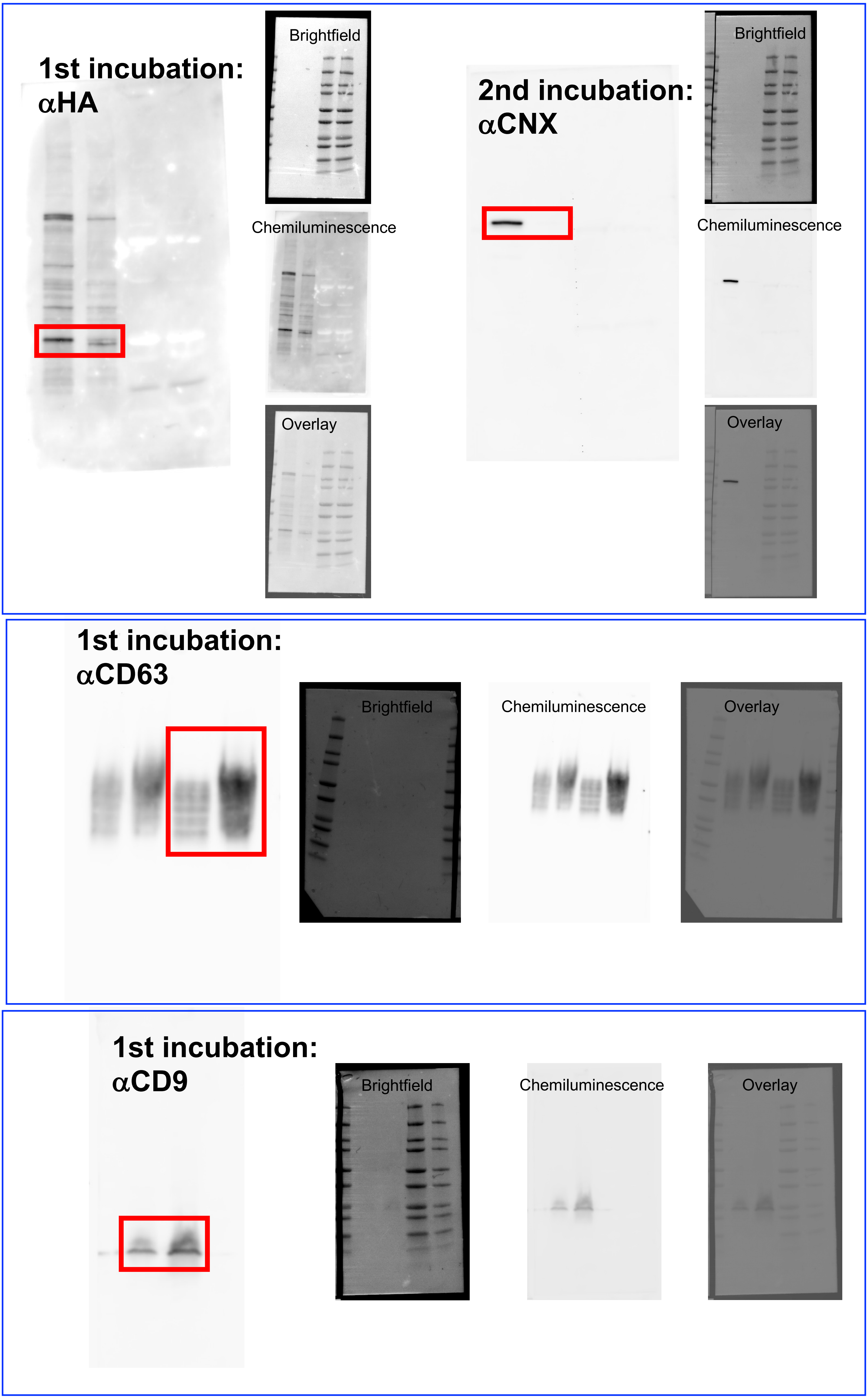

Figure 2B

3 blots were sequentially incubated with one or 2 different antibodies, in the indicated order. Raw brightfield (with colored protein molecular weight marker), chemiluminescence images as well as overlays are shown. The red box indicates the part of the blot included in the main figure.

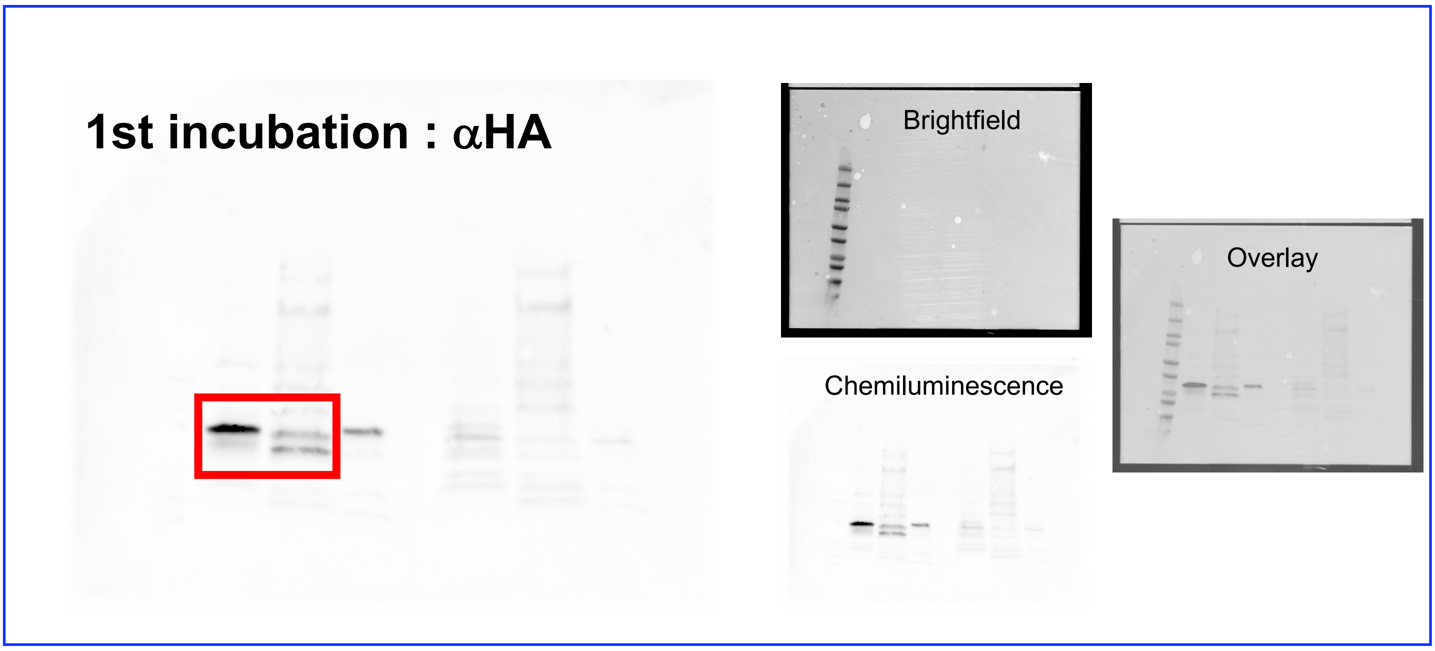

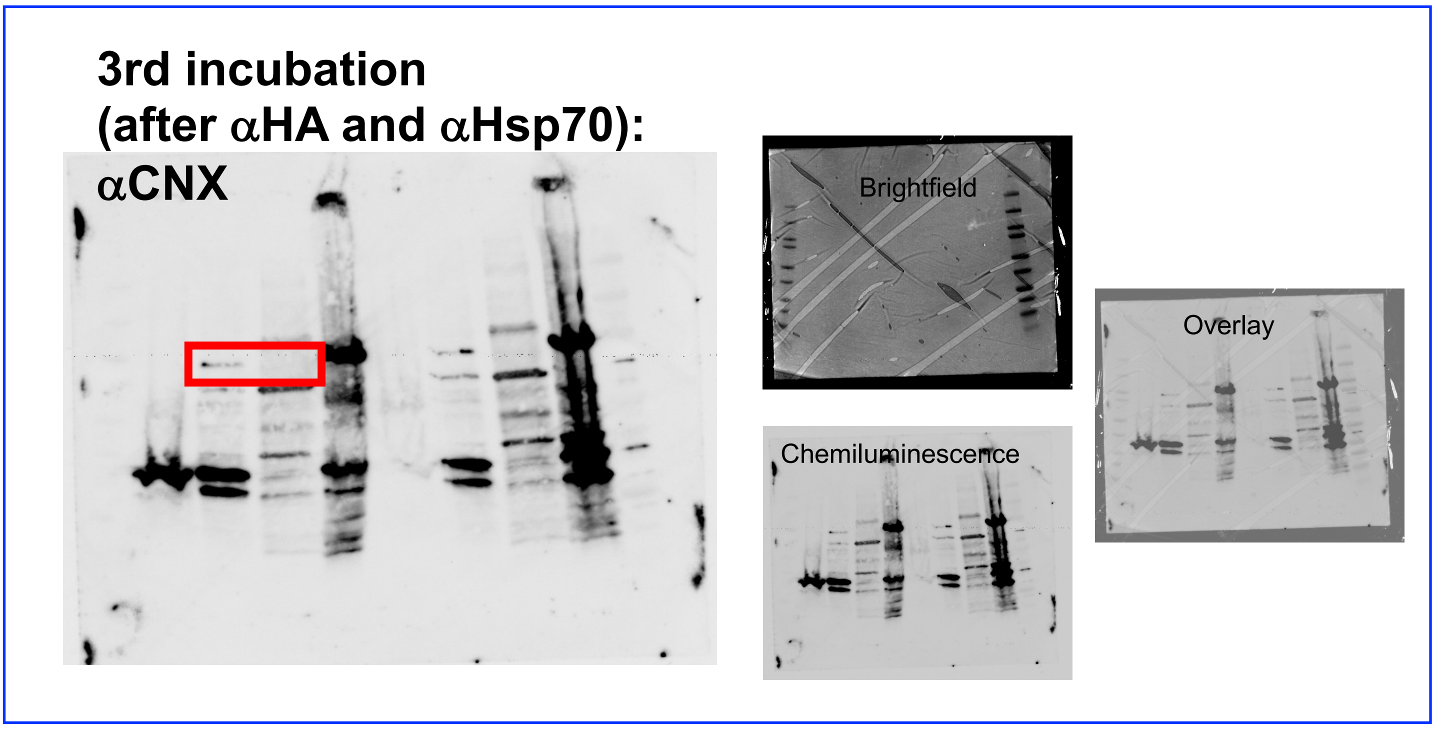

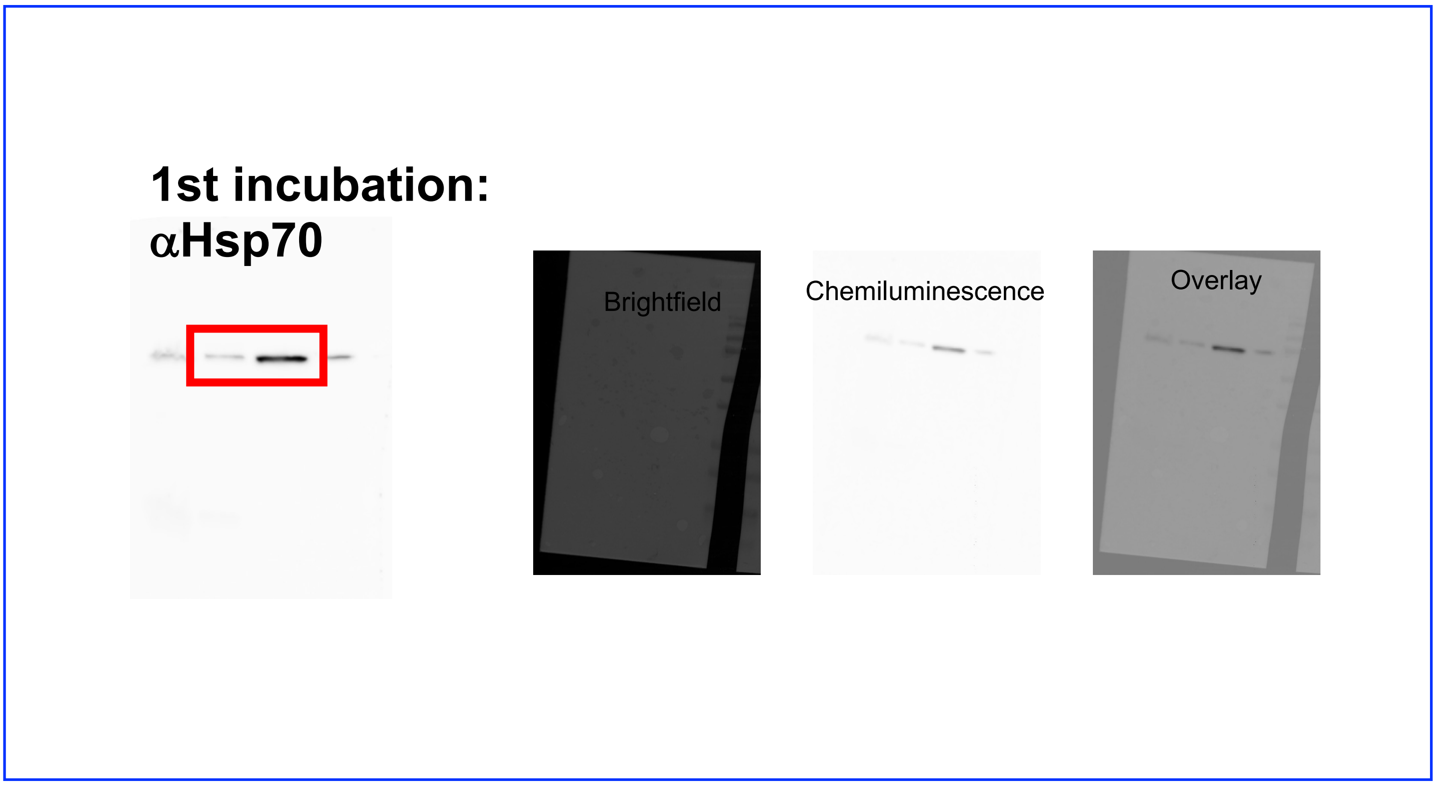

Figure 2D

2 blots were sequentially incubated with 2 different antibodies, in the indicated order. Raw brightfield (with colored protein molecular weight marker), chemiluminescence images as well as overlays are shown. The red box indicates the part of the blot included in the main figure.

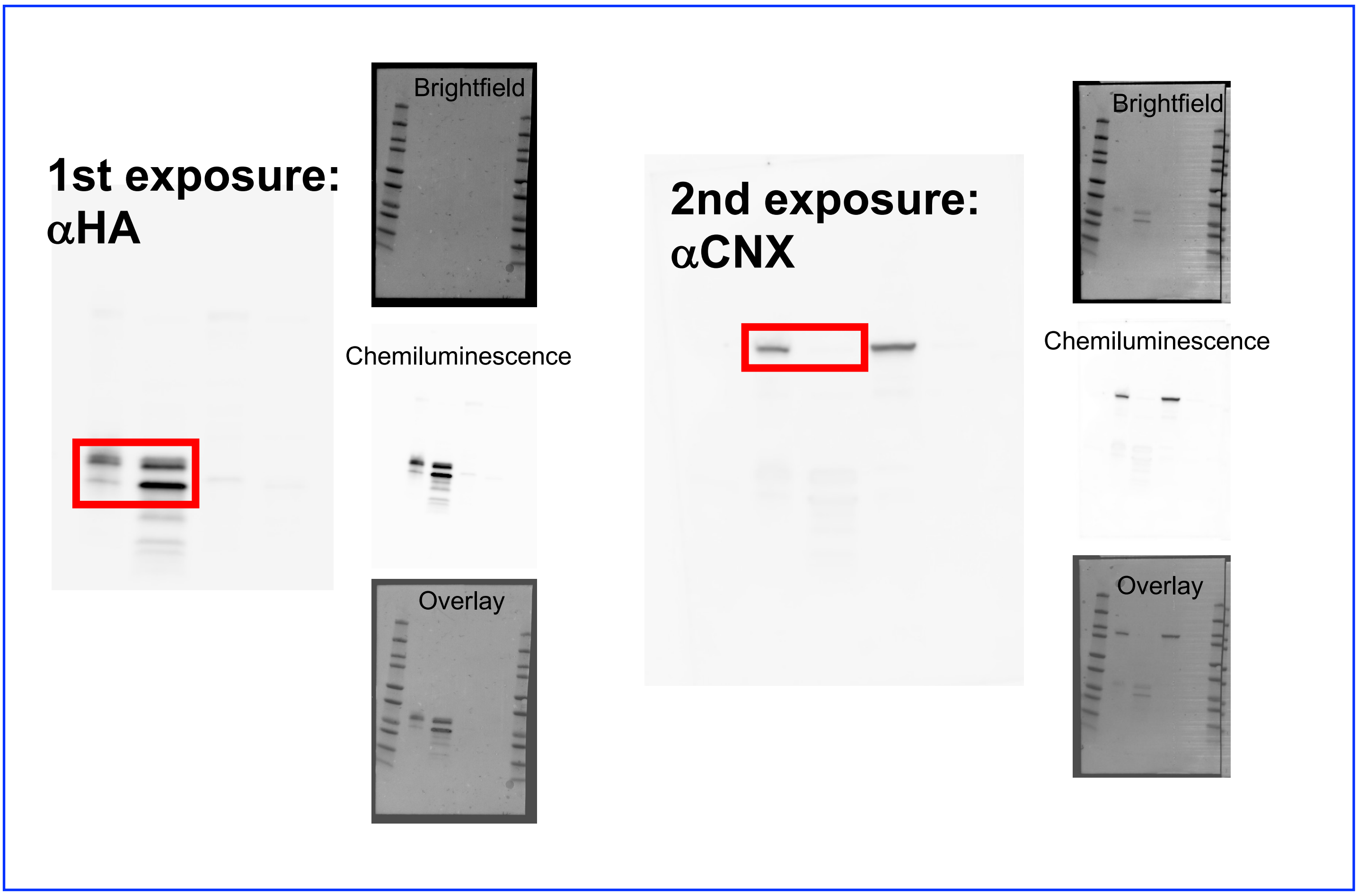

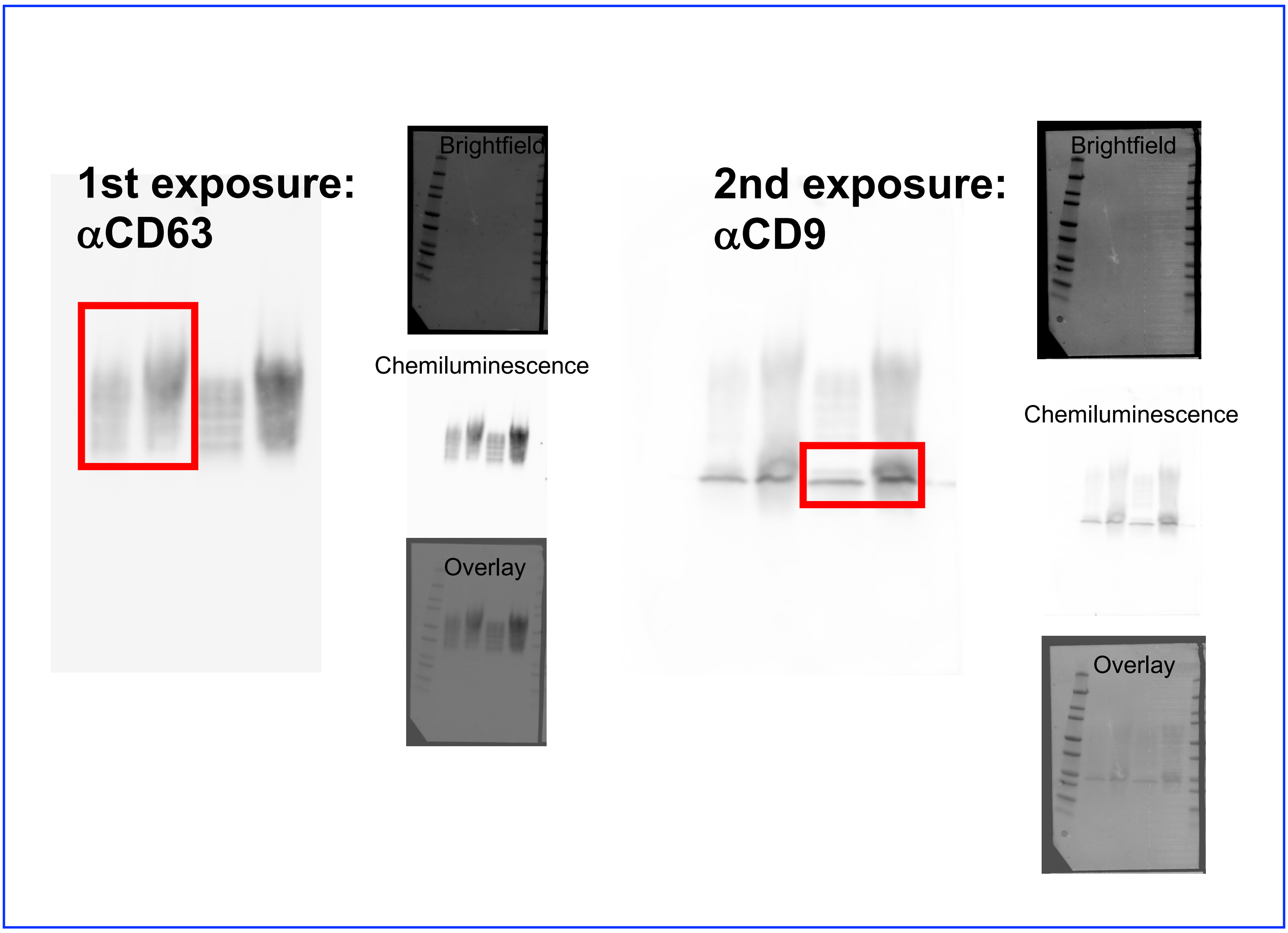

Figure 3A

3 blots were sequentially incubated with one or 2 different antibodies, in the indicated order. Raw brightfield (with colored protein molecular weight marker), chemiluminescence images as well as overlays are shown. The red box indicates the part of the blot included in the main figure.

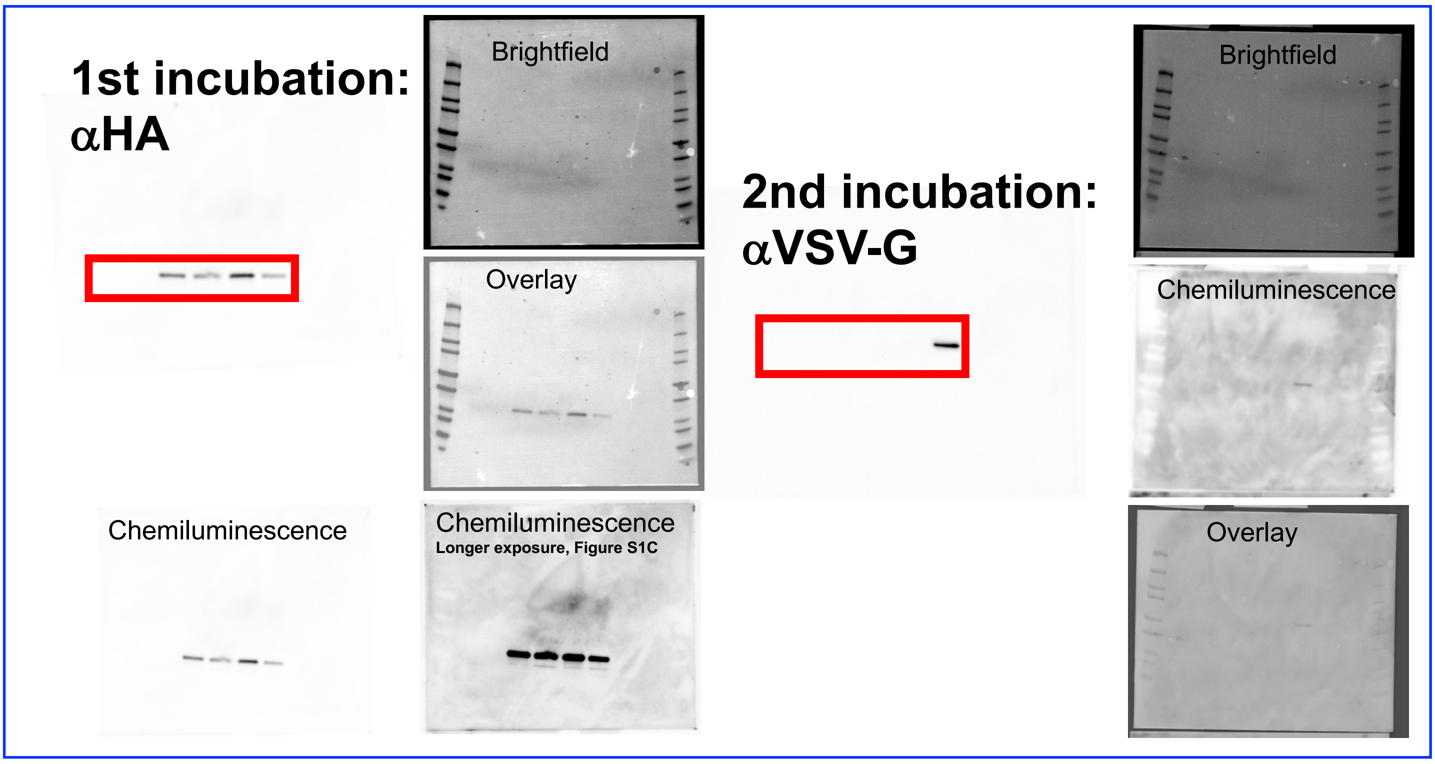

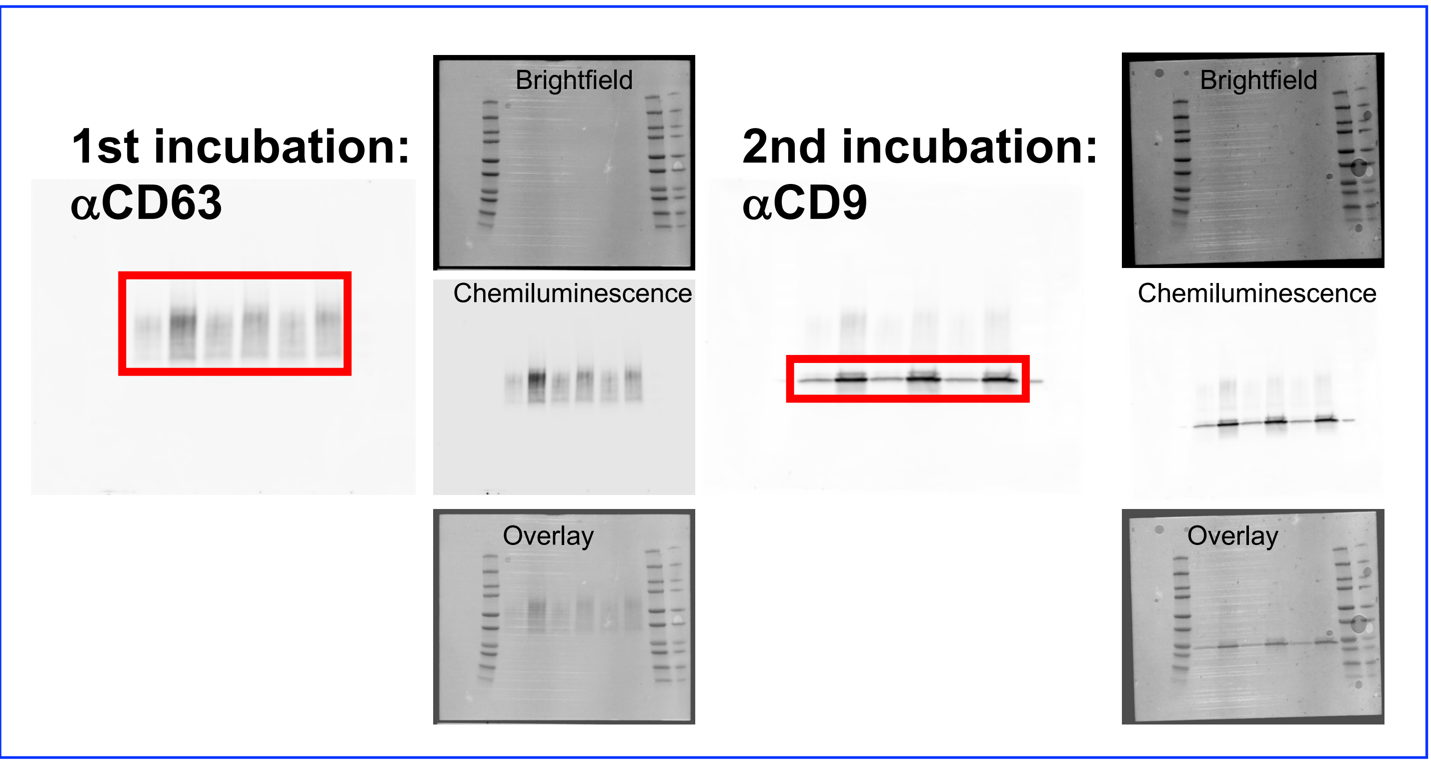

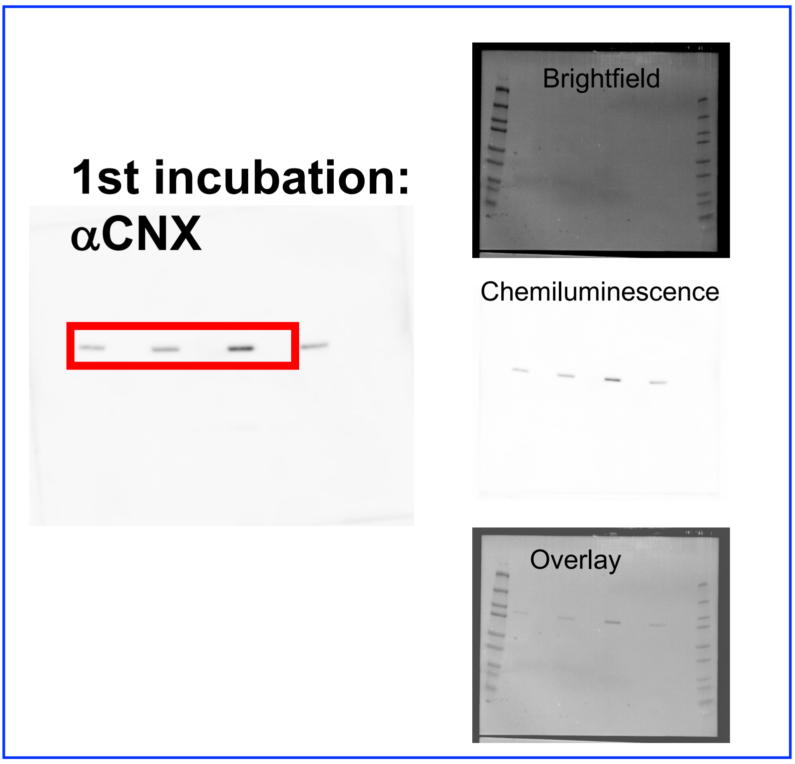

Figure S1A

One blot was incubated with one antibody. Raw brightfield (with colored protein molecular weight marker), chemiluminescence images as well as overlays are shown. The red box indicates the part of the blot included in the main figure.

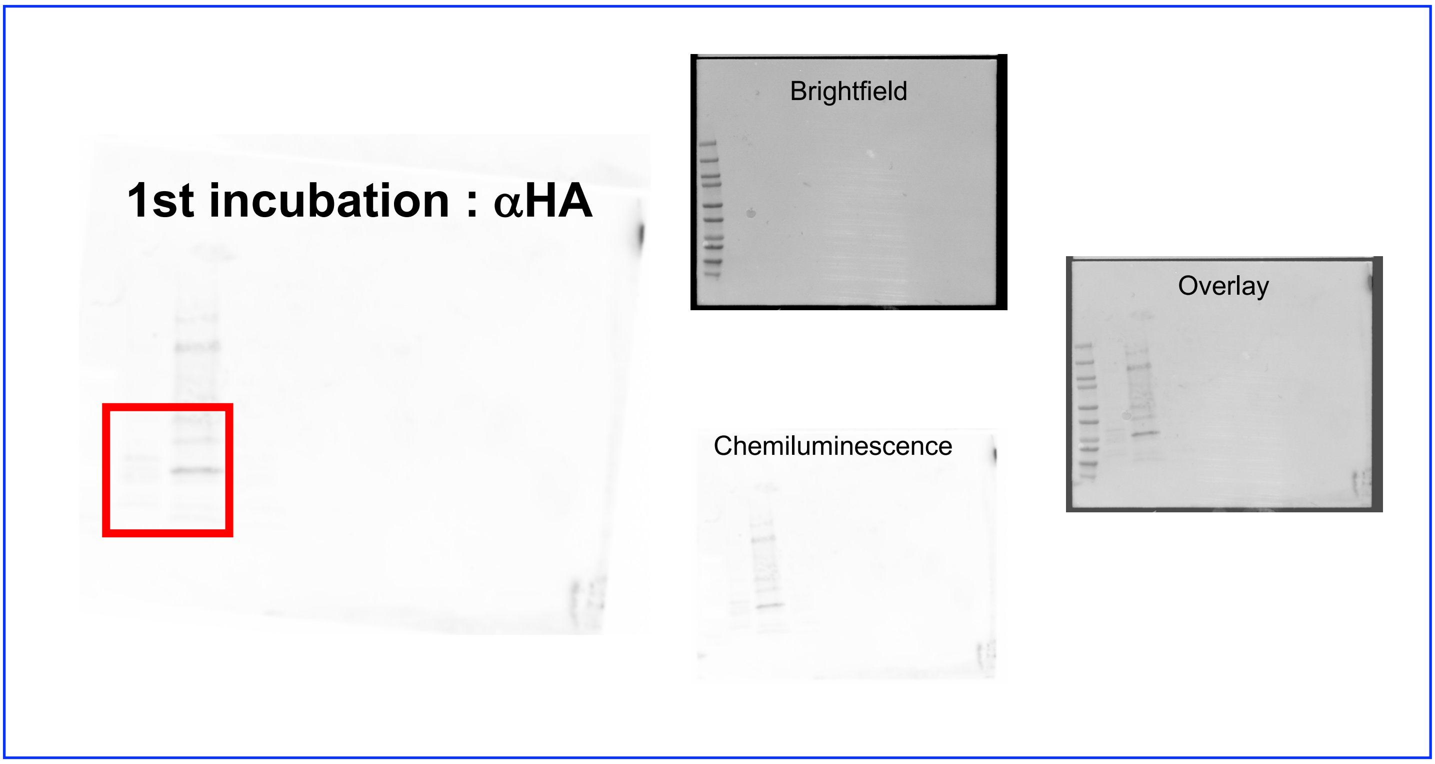

Supplement: Supplementary file 2 — Supplementary Information 2. [file 41598_2023_28306_MOESM2_ESM.pdf]
