# Supplementary material for: Efficient cell death mediated by bioengineered killer extracellular vesicles
Source: Sci Rep. 2023 Jan 19;13:1086. doi: 10.1038/s41598-023-28306-8 (PMC9852484; doi:10.1038/s41598-023-28306-8)
Supplement: Supplementary file 3 — Supplementary Information 3. [file 41598_2023_28306_MOESM3_ESM.pdf]

Figure S1. Killer EVs are potent in vitro.

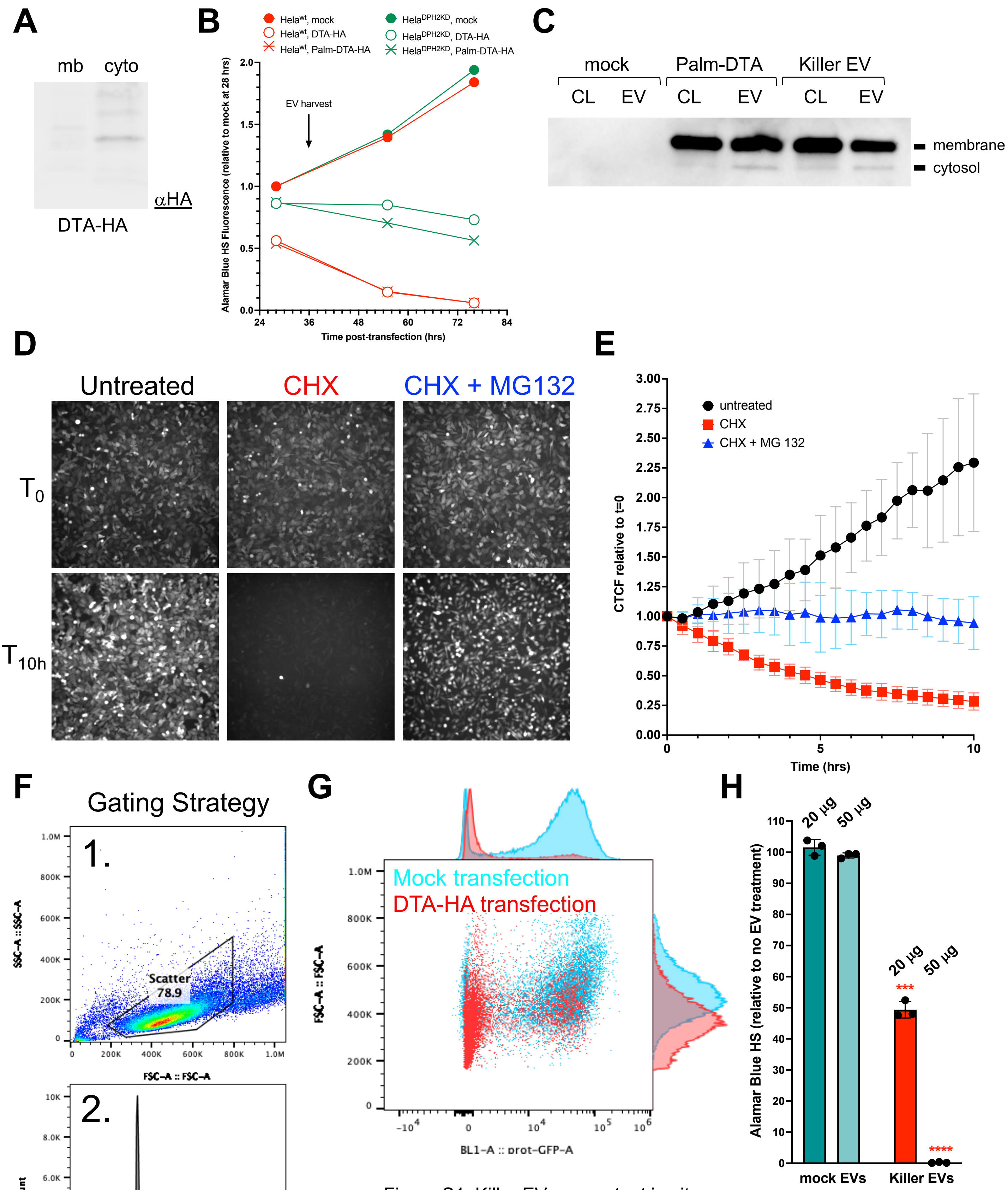

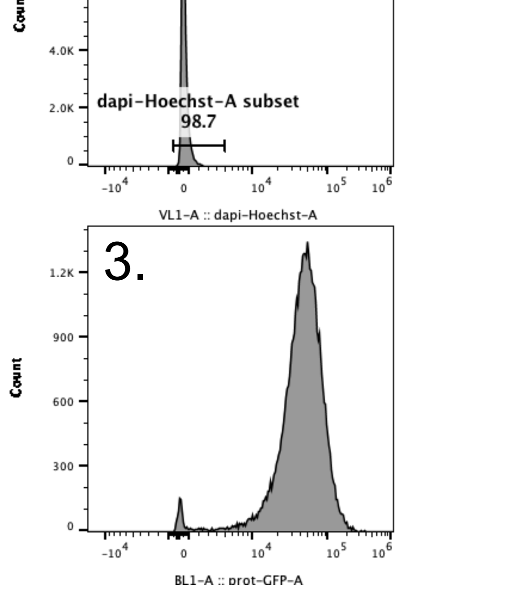

Figure S1. Killer EVs are potent in vitro.

A. Cytosol / membrane fractionation of DPH2KD cells expressing DTA-HA. B. Cell growth curve of parent and DPH2KD HeLa cells transfected with either mock, or DTA-HA, or Palm-DTA-HA. The time at which conditioned medium is harvested for EV preparation is indicated with an arrow. C. Longer exposure of the αHA western blot in Figure 3A. Highlighted are the palmitoylated and de-palmitoylated forms of Palm-DTA-HA that fractionate in the membrane or cytosolic fraction respectively (Figure 2B). D. Characterization of the GFP-PEST expressing HT1080 clone. Cells were either treated with the protein translation inhibitor 50 µg/mL cycloheximide (CHX, Cell Signaling, Massachusetts, U.S.A.), or with the proteasome inhibitor 50 µM MG132 (Merck Millipore, Massachusetts, U.S.A.), or left untreated and were imaged for the indicated time. Representative images are shown for each sample. E. Green fluorescence was measured over time and the Corrected Total Cell Fluorescence was plotted. F. Gating strategy used for FACS analysis. 1. A scatter gate was applied on the FSC/SSC plot. 2. A DAPI⁻ gate was then applied, to obtain the Green fluorescence plot in 3. G. GFP-PEST HT1080 cells are sensitive to DTA as transfection with a DTA-HA encoding plasmid virtually abolishes Green fluorescence in these cells after 24 hours of transfection. H. HeLa cells incubated with increasing doses (20µg or 50µg) of the indicated EVs for 3 days were submitted to a cell viability assay.
