# Supplementary material for: Efficient cell death mediated by bioengineered killer extracellular vesicles
Source: Sci Rep. 2023 Jan 19;13:1086. doi: 10.1038/s41598-023-28306-8 (PMC9852484; doi:10.1038/s41598-023-28306-8)
Supplement: Supplementary file 4 — Supplementary Information 4. [file 41598_2023_28306_MOESM4_ESM.pdf]

# Table S1. Plasmids used in this study

| Construct expressed | Plasmid name | Origin |
|---|---|---|
| GFP-PEST | pcDNA3.3_d2eGFP | Addgene, Cat # 26821 |
| NanoLuc-Hsp70 | NanoLuc-Hsp70 | Bonsergent et al. 2021 |
| DTA-HA | JDB10 | This study |
| mCherry | pSV40-mCherry-P2A-Hygro | Addgene, Cat # 106478 |
| Palm-DTA-HA | JDB27 | This study |
| VSV-G | pCMV-VSV-G | Addgene, Cat # 8454 |
| Mock (empty) | pCDNA3.1 | Invitrogen, Cat # V79020 |
